# Diagnostic accuracy of digital X-ray radiogrammetry on hand bone loss for patients with rheumatoid arthritis

**DOI:** 10.1097/MD.0000000000017280

**Published:** 2019-09-27

**Authors:** Hong-Jian An, Jun Zhang

**Affiliations:** a Department of Computed Tomography, Qishan County Hospital, Qishan, Shaanxi; b Department of Imaging, The Fourth People's Hospital of Shaanxi, Xi’an, China.

**Keywords:** digital X-ray radiogrammetry, hand bone loss, rheumatoid arthritis, sensitivity, specificity

## Abstract

**Background:**

This study will aim to evaluate the diagnostic accuracy of digital X-ray radiogrammetry (DXR) on hand bone loss (HBL) for rheumatoid arthritis (RA).

**Methods:**

In this study, we will search the literature from PubMed, EMBASE, Cochrane Library, PsycINFO, Web of Science, Google Scholar, Cumulative Index to Nursing and Allied Health Literature, Allied and Complementary Medicine Database, Chinese Biomedical Literature Database, China National Knowledge Infrastructure, and WANFANG from the inception to June 1, 2019 without language restrictions. All case–controlled studies on assessing diagnostic accuracy of DXR on HBL for diagnosis of RA will be included. Quality Assessment of Diagnostic Accuracy Studies tool will be used for eligible studies. We will apply RevMan V.5.3 software and Stata V.12.0 software for statistical analysis.

**Results:**

We will evaluate diagnostic accuracy of DXR on HBL in patients with RA by assessing the sensitivity, specificity, positive likelihood ratio, negative likelihood ratio, and diagnostic odds ratio.

**Conclusion:**

This study will detect the diagnostic accuracy of DXR evaluation on HBL in patients with RA.

**Systematic review registration:**

PROSPERO CRD42019139489.

## 1 Introduction

Rheumatoid arthritis (RA) is a chronic, autoimmune, systemic, and inflammatory disease.^[1,2]^ It has been estimated that such disorder affects about 0.5% to 1.0% of the adults,^[3,4]^ and about 5 to 50 new cases per 100,000 persons each year.^[3]^ It often occurs more in females than males with a ratio of 3:1.^[5]^ Such disorder is very common and often affects small joints of the hands and feet.^[6–9]^ Of those, hand bone loss (HBL) is often associated with progressive joint destruction of RA.^[10,11]^ Thus, it may predict the severity and progression of patients with RA.

Digital X-ray radiogrammetry (DXR) is a technique that has been important role in diagnosis of HBL in patients with RA.^[11,12–15]^ In addition, previous clinical studies have reported the diagnostic accuracy of DXR on HBL in patients with RA.^[11,16–18]^ However, no study has explored its diagnostic accuracy on HBL in patients with RA based on the evidence-based medicine levels. Thus, this study will systematically investigate the diagnostic accuracy of DXR on HBL in patients with RA.

## 2 Methods

### 2.1 Study protocol registration

This study has been registered via PROSPERO CRD42019139489. It has been carried out to follow the guideline of Preferred Reporting Items for Systematic Reviews and Meta-Analysis Protocol (PRISMA-P) statement.^[19]^

### 2.2 Eligibility criteria for study selection

#### 2.2.1 Type of studies

We will include case–controlled studies reporting the diagnostic accuracy of DXR on HBL in patients with RA.

#### 2.2.2 Type of participants

In this study, reporting on individuals with RA will be included without restrictions of race, gender, and age.

#### 2.2.3 Type of index test

Index test: DXR evaluation on HBL will be used to diagnose patients with RA. However, we will exclude patients who received both DXR and other tests.

Reference test: patients with standard diagnosis of American College of Rheumatology or European League Against Rheumatism, or guideline of Chinese rheumatoid arthritis treatment will be included.

#### 2.2.4 Type of outcome measurements

In this study, we will use sensitivity and specificity as primary outcome measurements. We will utilize positive likelihood ratio, negative likelihood ratio, and diagnostic odds ratio as secondary outcome measurements.

### 2.3 Literature records selection

#### 2.3.1 Electronic searches

PubMed, EMBASE, Cochrane Library, PsycINFO, Web of Science, Google Scholar, Cumulative Index to Nursing and Allied Health Literature, Allied and Complementary Medicine Database, Chinese Biomedical Literature Database, China National Knowledge Infrastructure, and WANFANG databases will be used to search a comprehensive relevant record from the inception to June 1, 2019 without language restrictions. A comprehensive literature search strategy for PubMed is presented in Table 1. We will also adapt similar literature search strategy for other electronic databases.

**Table 1:**
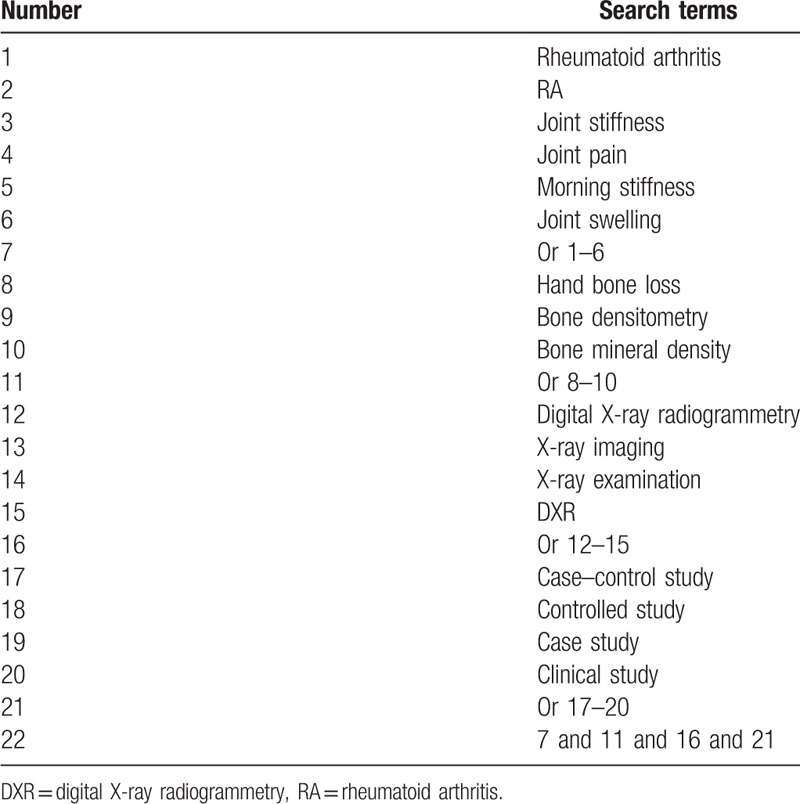
Search strategy used in PubMed database.

#### 2.3.2 Other resources

Grey literature will also be searched, such as conference proceedings, dissertations, and reference list of relevant reviews.

### 2.4 Data collection and analysis

#### 2.4.1 Selection of studies

**Figure 1:**
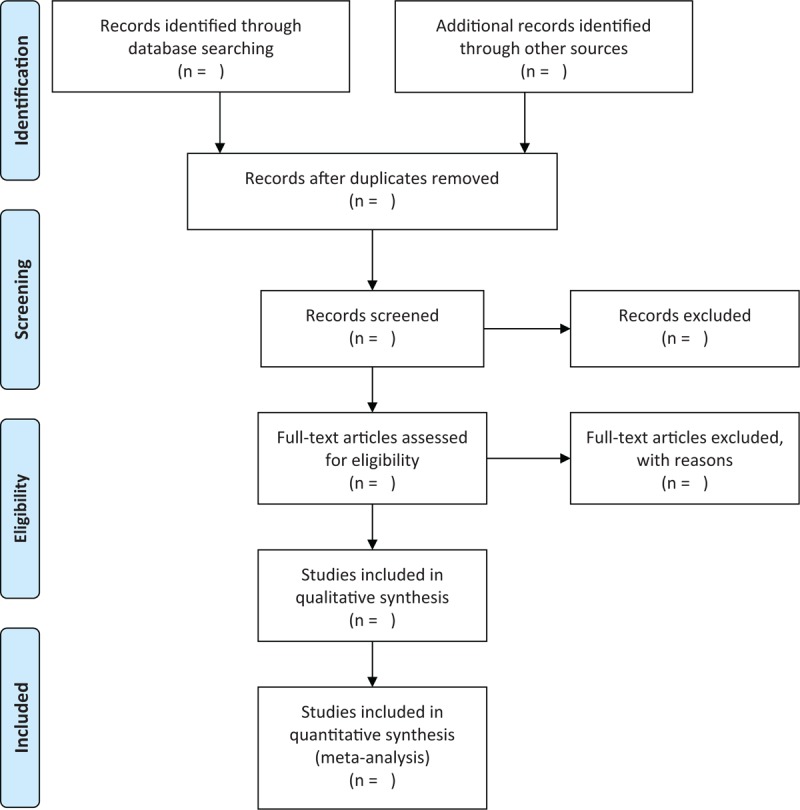
Flow diagram of study selection process.

Two reviewers will independently select titles and abstracts firstly based on the previously defined eligibility criteria. Any differences between 2 reviewers will be solved. All irrelevant studies will be excluded after initial selection. Then, all rest papers will be recorded to check if they meet final eligibility criteria. We will present the results of study selection in PRISMA flowchart in Figure 1.

#### 2.4.2 Data collection

Two reviewers will independently collect the data using predefined data extraction sheet. Any disagreements will be resolved by discussion with the help of a third reviewer. The data collection consist of title, authors, publication date, location, study design, sample size, index test, reference test, sensitivity, specificity, positive likelihood ratio, negative likelihood ratio, and funding information.

#### 2.4.3 Dealing with missing data

We plan to inquire the data from the authors of primary studies if they are missing and insufficient. We will only analyze available data if that data is not achievable.

### 2.5 Methodological quality assessment

We will use Quality Assessment of Diagnostic Accuracy Studies tool to check methodological quality assessment.^[20]^ Two reviewers will independently evaluate the methodological quality for each eligible study. Any disagreements regarding methodological quality evaluation will be solved by discussion with a third reviewer.

### 2.6 Statistical analysis

We will apply RevMan V5.3 and Stata V.12.0 softwares (London, UK) for statistical analysis. We will calculate descriptive statistics and 95% confidence intervals for each primary study. We will calculate the pooled sensitivity, specificity, positive likelihood ratio, negative likelihood ratio, and diagnostic odds ratio. We will derive descriptive forest plot and a summary receiver operating characteristic plot will be carried out.

#### 2.6.1 Assessment of heterogeneity

We will assess heterogeneity using *I*^2^ statistic. *I*^2^ ≤ 50% indicates low heterogeneity, while *I*^2^ > 50% indicates significant heterogeneity.

#### 2.6.2 Data synthesis

We will conduct meta-analysis if heterogeneity is low (*I*^2^ ≤ 50%). Otherwise, we will carry out subgroup analysis, and meta-analysis will be performed based on the results of subgroup analysis if heterogeneity is significant (*I*^2^ > 50%). If there is still substantial heterogeneity after subgroup analysis, meta-analysis will not be carried out. Then, we will use bivariate random-effects regression approach to estimate sensitivity and specificity.

#### 2.6.3 Subgroup analysis

Subgroup analysis will be performed to check possible factors that may lead to the significant heterogeneity according to the different characteristics, treatments, and comparators.

#### 2.6.4 Sensitivity analysis

Sensitivity analysis will be carried out by removing low methodological quality studies.

#### 2.6.5 Reporting bias

We will conduct funnel plots to investigate the possible reporting biases among included studies.^[21]^

### 2.7 Ethics and dissemination

We will not inquire individual patient data, thus no research ethic approval is needed. We expect to publish results of this study at peer-reviewed journals.

## 3 Discussion

Previous studies have reported that DXR can be used to predict and diagnosis HBL for patients with RA.^[11,16–18]^ However, diagnostic accuracy of DXR on HBL in patients with RA still fails to support on the levels of evidence-based medicine. Thus, this study will systematically investigate the diagnostic accuracy of DXR on HBL in patients with RA. The results of this study will provide a summary of the up-to-date evidence on the diagnostic accuracy of DXR on HBL in patients with RA on evidence-based medicine levels. It will also help to predict patients with RA at early stage.

## Author contributions

**Conceptualization:** Hong-Jian An, Jun Zhang.

**Data curation:** Hong-Jian An, Jun Zhang.

**Formal analysis:** Hong-Jian An.

**Funding acquisition:** Jun Zhang.

**Investigation:** Jun Zhang.

**Methodology:** Hong-Jian An.

**Project administration:** Jun Zhang.

**Resources:** Hong-Jian An.

**Software:** Hong-Jian An.

**Supervision:** Jun Zhang.

**Validation:** Hong-Jian An, Jun Zhang.

**Visualization:** Hong-Jian An, Jun Zhang.

**Writing – original draft:** Hong-Jian An, Jun Zhang.

**Writing – review & editing:** Hong-Jian An, Jun Zhang.

## References

[1] Deane KD Holers VM The natural history of rheumatoid arthritis. Clin Ther 2019;41:1256–69. 31196652 10.1016/j.clinthera.2019.04.028

[2] Conigliaro P Triggianese P De Martino E Challenges in the treatment of rheumatoid arthritis. Autoimmun Rev 2019;18:706–13. 31059844 10.1016/j.autrev.2019.05.007

[3] Silman AJ Pearson JE Epidemiology and genetics of rheumatoid arthritis. Arthritis Res 2002;4 Suppl 3:S265–72. 12110146 10.1186/ar578 PMC3240153

[4] Scott DL Wolfe F Huizinga TW Rheumatoid arthritis. Lancet 2010;376:1094–108. 20870100 10.1016/S0140-6736(10)60826-4

[5] Jawaheer D Lum RF Gregersen PK Criswell LA Influence of male sex on disease phenotype in familial rheumatoid arthritis. Arthritis Rheum 2006;54:3087–94. 17009227 10.1002/art.22120

[6] Minnock P FitzGerald O Bresnihan B Women with established rheumatoid arthritis perceive pain as the predominant impairment of health status. Rheumatology (Oxford) 2003;42:995–1000. 12730516 10.1093/rheumatology/keg281

[7] Gonzalez Gay MA Gonzalez Juanatey C Martin J Rheumatoid arthritis: a disease associated with accelerated atherogenesis. Semin Arthritis Rheum 2005;35:8–17. 16084219 10.1016/j.semarthrit.2005.03.004

[8] van der Heijde DM Radiographic imaging: the “gold standard” for assessment of disease progression in rheumatoid arthritis. Rheumatology (Oxford) 2000;39 Suppl 1:9–16. 11001374 10.1093/oxfordjournals.rheumatology.a031496

[9] Brower AC Use of the radiograph to measure the course of rheumatoid arthritis. The gold standard versus fool's gold. Arthritis Rheum 1990;33:316–24. 2180404 10.1002/art.1780330303

[10] de Rooy DP Kälvesten J Huizinga TW Loss of metacarpal bone density predicts RA development in recent-onset arthritis. Rheumatology (Oxford) 2012;51:1037–41. 22258391 10.1093/rheumatology/ker435

[11] Forslind K Boonen A Albertsson K Hand bone loss measured by digital X-ray radiogrammetry is a predictor of joint damage in early rheumatoid arthritis. Scand J Rheumatol 2009;38:431–8. 19922017 10.3109/03009740902939376

[12] Bottcher J Pfeil A Heinrich B Digital radiogrammetry as a new diagnostic tool for estimation of disease-related osteoporosis in rheumatoid arthritis compared with pQCT. Rheumatol Int 2005;25:457–64. 15761729 10.1007/s00296-004-0560-z

[13] Hoff M Haugeberg G Odegard S Cortical hand bone loss after 1 year in early rheumatoid arthritis predicts radiographic hand joint damage at 5-year and 10-year follow-up. Ann Rheum Dis 2009;68:324–9. 18339664 10.1136/ard.2007.085985

[14] Kapetanovic MC Lindqvist E Algulin J Early changes in bone mineral density measured by digital X-ray radiogrammetry predict up to 20 years radiological outcome in rheumatoid arthritis. Arthritis Res Ther 2011;13:R31. 21345204 10.1186/ar3259 PMC3241375

[15] Forslind K Kalvesten J Hafstrom I Svensson B BARFOT Study Group. Does digital X-ray radiogrammetry have a role in identifying patients at increased risk for joint destruction in early rheumatoid arthritis? Arthritis Res Ther 2012;14:R219. 23068060 10.1186/ar4058 PMC4060357

[16] Rezaei H Saevarsdottir S Geborek P Evaluation of hand bone loss by digital X-ray radiogrammetry as a complement to clinical and radiographic assessment in early rheumatoid arthritis: results from the SWEFOT trial. BMC Musculoskelet Disord 2013;14:79. 23497111 10.1186/1471-2474-14-79 PMC3599105

[17] Dirven L Güler-Yüksel M de Beus WM Changes in hand bone mineral density and the association with the level of disease activity in patients with rheumatoid arthritis: bone mineral density measurements in a multicenter randomized clinical trial. Arthritis Care Res (Hoboken) 2011;63:1691–9. 21905248 10.1002/acr.20612

[18] Güler-Yüksel M Klarenbeek NB Goekoop-Ruiterman YP Accelerated hand bone mineral density loss is associated with progressive joint damage in hands and feet in recent-onset rheumatoid arthritis. Arthritis Res Ther 2010;12:R96. 20482894 10.1186/ar3025 PMC2911882

[19] Shamseer L Moher D Clarke M PRISMA-P Group. Preferred reporting items for systematic review and meta-analysis protocols (PRISMA-P) 2015: elaboration and explanation. BMJ 2015;349:g7647. 10.1136/bmj.g7647 25555855

[20] Whiting PF Rutjes AW Westwood ME QUADAS-2 Group. QUADAS-2: a revised tool for the quality assessment of diagnostic accuracy studies. Ann Intern Med 2011;155:529–36. 22007046 10.7326/0003-4819-155-8-201110180-00009

[21] Deeks JJ Macaskill P Irwig L The performance of tests of publication bias and other sample size effects in systematic reviews of diagnostic test accuracy was assessed. J Clin Epidemiol 2005;58:882–93. 16085191 10.1016/j.jclinepi.2005.01.016

